# Dr. Walker, in Answer to Eosaco

**Published:** 1805-06-01

**Authors:** 


### *To the Editors of the Medical and Physical Journal.*

GENTLEMEN, *Salisbury Square*, 2, *v*. 1805.

THE "literary assassin," may screen himself under the veil of a fictitious signature, (vide Medical and Chirurgical Review, July 1804); or by withholding all manner of subscription (id. March 1803). The Philologos and Eosaco of your late numbers, are characters of a very different description. It is pleasant even to reply to the ingenuous unknown. I have not a doubt but the case mentioned by Eosaco, in 399, was a complete case of vacciolation; and the deep hole in the centre, whence a considerable quantity of pus was discharged, must only have been produced by a rather heavy application of the lancet at the time of the

 inoculation.

inoculation. Such appearances of irregularity very frequently meet my eye; on which occasions I immediately remove the scab, wipe out the subjacent mass of pus, (enough of it to produce on one and twenty subjects a completely spurious, if any, effect by inoculation) and then, cutting up the vascular ring which bounded it, obtain only the true vacciolous bunoserous, Jennerian, or guardian fluid.

My unwavering confidence in the above conclusion arises from the very circumstantial statements of Eosaco, both as to the facts, and the time of their happening. Blood was drawn at the time of inoculation; this was by too heavy an application of the lancet. Yet the matter was not washed completely away; some minute particles of it were received by the cellulæ, or loculi *sub cuticula*, or by minute ramifications of absorbents, or veins, or nerves, or whatever else they may be which are first acted upon, and which, accordingly as they may have been cut or wounded by the instrument, may have immediately laid hold of the virus, or may for a time have collapsed, or become paralysed, producing those varieties in point of time and appearance, which now and then occur. The appearance on the eighth day was such as proved, that genuine matter had been applied, and that the perfect effect had been produced. Had the inoculation been performed lightly, the pock would not yet have been so large, and would have had a more transparent appearance; the areola might also have been rather later, but not more perfect in its appearance. The characteristic scab would have filled up the central part of the pock, which pus, producing successively the yellow and green appearances, induced by the wound from the lancet, had completely occupied. The scab would have exhibited the gradations of shade from yellow to dark brown; it would not have cracked, but, thickest at its centre, would at length have turned out, or fallen off, firm as a tamarind stone.

How happens it that the lancet thus sometimes produces, besides the proposed effect (infection) so great a collection of pus in the centre of the pock? A similar wound in an ordinary way would not be greater in its effects than, in dress, the female very commonly experiences from a scratch of a pin, the man from a scratch of his razor? Does the virus introduced prevent the ordinary healing process being set up as on the surface, by giving the circumjacent parts a different action to occupy them with; and does the healing process hereby become a more deeply-seated business with formation of pus, &c. burrowing deeper sometimes than the pit which that congeries of cells, the whole substance stance of the vacciolous pock, does produce and occupy, invariably leaving the indelible cicatrix.

The following extracts, from a letter of Dr. Jenner, must be acceptable to many of your readers.

J. W.

"Dear Sir,

"I have enclosed a few ivory points, coated with vaccine virus. It is just taken from a fine eight-day pustule, and I could wish you to infect two or three children with it. I am introducing no stranger to you; but a branch of that friend of humanity with which you became first acquainted in the Mediterranean. In short, it is from my old stock, which we have been constantly using here from the year 1799. It has been in constant use in my own immediate hands from December last. One of my principal motives for wishing to see it revived at the Central House is, that any gentleman may have an opportunity of witnessing the permanency (with respect to its qualities) of the vaccine fluid for six successive years. During this time, it has been constantly passing from one human being to another. You will not perceive any difference in the pustules excited by this and that you are now in the habit of using.

"My paper on Herpes is unpopular, I find; I am sorry for it. But for all this, I hope you will bear an eye to it. Indeed you have already borne witness to those facts inculcated by me long before my departure from town.

"A very curious and interesting case has lately occurred; you shall have the outline.

"A child was inoculated for the small pox; the arm inflamed and *festered* to a considerable extent. Some time after, she was again inoculated with small-pox virus, and the result was not unlike the former inoculation. After the lapse of a considerable length of time, she was inoculated with active vaccine matter. The arm inflamed to a great extent, and a large pustule was quickly formed, but wanting in its perfect character. I was consulted as to the safety of the child, and of course gave an opinion that it was still insecure. On inquiry, I found it had been affected with herpetic blotches, almost from its earliest infancy. I recommended a remedy for the malady (which was applied with effect) and then a reinoculation with the vaccine fluid. The spell was broken (if I may use the expression) and now the vaccine sprung up, unimpeded and perfect in all its characters. Observe, during the presence of herpes, there may be no imperfection in the vaccine pock, and and there may be, and there certainly is, every gradation between that slight deviation which is of no sort of consequence, up to that which completely denies all security.

"Yours, very faithfully,

*Berkeley, April* 25, 1803. "EDWARD JENNER."

