# Addendum et Corrigendum

**Published:** 1805-06-01

**Authors:** 


### ADDENDUM ET CORRIGENDUM.

The following postscript to Dr. WALKER's letter, p. 541, came too late for insertion in its proper place.

"'P. S. To the above extracts, which I have taken the liberty of sending you for public good, without waiting the permission of Dr. Jenner, and for which I trust he will pardon me, I can now add, from a later letter, another fact on the very curious and interesting case."—'The child having herpes was put to bed to her brother, labouring under the small-pox; but still it remained proof against the infection, both of the small-pox and cow-pock, until the herpes disappeared, which had continued from the first till the ninth year of her age.

"P. 540, l. 20, for time *and,* read time *of* appearance,"

